# Sequence Characterization and Spatiotemporal Expression Patterns of *PbS*_***26***_ *-RNase* Gene in Chinese White Pear (*Pyrus bretschneideri*)

**DOI:** 10.1155/2014/147061

**Published:** 2014-03-05

**Authors:** Lin Zhang, Baoguang Jia, Feng Zou, Xiaofeng Tan, Min Liu, Zhibo Song, Yanling Zeng, Nan Jiang, Deyi Yuan

**Affiliations:** ^1^Key Laboratory of Cultivation and Protection for Non-Wood Forest Trees, Ministry of Education, Central South University of Forestry and Technology, Changsha 410004, China; ^2^Department of Plant Science and Landscape Architecture, University of Connecticut, Storrs, CT 06269, USA

## Abstract

Many flowering plants exhibit an important intraspecific reproductive barrier phenomenon, that is, self-incompatibility (SI), in which *S-RNase* genes play a significant role. To clarify the specific function of *S-RNase* genes in Chinese pears, the full length cDNA of *PbS*_*26*_ *-RNase* was isolated by rapid amplification of cDNA ends (RACE) technology from Chinese white pear (*Pyrus bretschneideri*) cultivar “Hongpisu.” The cDNA sequence for *PbS*_*26*_ *-RNase* was deposited in GenBank under accession number EU081888. At the amino acid level, the *PbS*_*26*_ *-RNase* displayed the highest similarity (96.9%) with PcSa-RNase of *P. communis*, and only seven amino acid differences were present in the two S-RNases. Phylogenetic analysis of rosaceous S-RNases indicated that the *PbS*_*26*_ *-RNase* clustered with maloideous S-RNases, forming a subfamily-specific not a species-specific group. The *PbS*_*26*_ *-RNase* gene was specifically expressed in the style but not other tissues/organs. The expression level of the *PbS*_*26*_ *-RNase* gene rapidly increased at bell balloon stage (BBS), and then it dropped after pollination. However, the abundance of the *PbS*_*26*_ *-RNase* gene transcript in the style was greater after cross-pollination than after self-pollination. In addition, a method for rapidly detecting the *PbS*_*26*_ *-RNase* gene was developed via allele-specific primers design. The present study could provide a scientific basis for fully clarifying the mechanism of pear SI at the molecular level.

## 1. Introduction

In flowering plants (also called angiosperms), an intraspecific reproductive mechanism, self-incompatibility (SI), has so far been widely known. This mechanism can prevent inbreeding and promote outcrossing [1]. One type of the SI, gametophytic SI (GSI), mainly exists in three plant families including Solanaceae, Scrophulariaceae, and Rosaceae, and it is controlled by a multiallelic S-locus [1–3]. The S-locus harbors a series of S-alleles that are divided into two types, that is, female S-genes and male S-genes. The expression products of the S-allele in the female organ (style) are S-RNases, a class of polymorphic proteins with ribonuclease activity.

Many fruit trees of Rosaceae, including apple, pear, and almond, belong to GSI-type plants. Among rosaceous members, Japanese pear (*Pyrus pyrifolia*) is the first species in which pistil S-allele specific proteins were successfully identified as S-RNases [3]. Afterwards, cDNAs encoding seven S-RNases (S_1_ to S_7_) of Japanese pear were cloned with their primary structure being determined [4, 5]. A hypervariable (HV) region, possibly associated with S-allele specific recognition, was identified [5]. Based on sequence characterization of Japanese pear *S-RNase *genes, a PCR-restriction fragment length polymorphism (PCR-RFLP) system was established for identifying S-genotypes of cultivars and searching new S-alleles [5]. Using this system, four additional alleles S_8_, S_9_, S_10_, and S_k_ were discovered and cloned, and accordingly the system was modified [6–8].

Pears native to China include four main cultivated species, that is, Chinese sand pear (*P. pyrifolia*), Chinese white pear (*P. bretschneideri)*, Ussurian pear (*P. ussuriensis*), and Xinjiang pear (*P. sinkiangensis*). Chinese pears were considered to be the major origin of Asian pears [9]. Knowledge of S-genotype is essential for pear production and breeding programs. The conventional pollination-based method for cultivars S-genotyping is time consuming and labor intensive. Moreover, the obtained results are often ambiguous due to environmental and physiological factors. PCR-based molecular techniques, such as PCR-RFLP and direct sequencing of amplified fragments, provide a rapid, reliable, and easily manipulated approach for pear cultivars S-genotyping. To date, a series of S-alleles have been found in Chinese pears and approximately 100 cultivars have been S-genotyped [10–15]. Of these S-alleles, only three full length cDNAs encoding S_13_-, S_16_-, and S_34_-RNases were reported and analyzed for their possible function in SI reaction [16, 17]. More recently, several pollen specific polymorphic F-box genes were isolated and considered to be the candidates of male S-genes in pears [18–24]. To fully clarify the mechanism of pear SI, it is necessary to investigate the interaction of the *S-RNase *genes with the male S-genes.

In this study we cloned and characterized the full length of cDNA of the* PbS*_*26*_ *-RNase *gene in *P. bretschneideri*. We studied the spatiotemporal expression patterns of this gene and compared its transcript abundance between incompatible pollination and compatible pollination. In addition, we developed a method for rapidly and accurately detecting the *PbS*_*26*_ *-RNase* gene.

## 2. Materials and Methods

### 2.1. Plant Materials

Plant materials used in this study include *P. bretschneideri* cultivars “Hongpisu” (S_12_S_26_) [13], “Hongxiangsu,” “Jinhua” (S_13_S_18_) [25], and 46 seedlings derived from cross of “Hongpisu” × “Jinhua” which are planted at Pear Germplasm Repository of Central South University of Forestry and Technology, Zhuzhou city, Hunan province. For cDNA cloning of *PbS*_*26*_ *-RNase *gene, styles at bell stage (BS) were collected from “Hongpisu.” For expression analysis of the *PbS*_*26*_ *-RNase* gene, styles of “Hongpisu” were collected at 96 h before anthesis (BA), 48 h BA, bell balloon stage (BBS), 12 h after pollination (AP), and 24 h AP, respectively. Moreover, stamens, roots, tender stems, and young leaves were also sampled for expression detection of the *PbS*_*26*_ *-RNase* gene. The plant materials were sampled and immediately frozen in liquid nitrogen and stored at −80°C until used.

### 2.2. Isolation of Nucleic Acids

Genomic DNA was isolated from young leaves with a CTAB method as described in our previous study [22]. Total RNA was extracted using Micro-to-Midi Total RNA Purification System (Invitrogen) and following the manufacturer's protocols. The quality of DNA and RNA was checked by electrophoresis in 1.0% TAE agarose gel.

### 2.3. Isolation of Full Length cDNA of *PbS*_***26***_ *-RNase* Gene

The full length cDNA of the *PbS*_*26*_ *-RNase* was amplified using 3′ RACE System for Rapid Amplification of cDNA Ends (Invitrogen, Version E). Total RNA was reverse-transcribed using SuperScript II Reverse Transcriptase with Adapter Primer provided in the system under condition of 10 min at 70°C, 50 min at 42°C, 15 min at 70°C, and 20 min at 37°C. The second PCR amplification was conducted with primers PF2 (5′- TGCCTCGCTCTTGAACAAA-3′) and AUAP. The PF2 anneals to 5′ UTR of most of pear S-RNases and was used for pear S-RNase cloning [6, 16]. The AUAP primer was provided in the 3′ RACE system. The amplification program consisted of 35 cycles of 30 s at 94°C, 30 s at 59°C, 3 min at 72°C, and a final extension of 7 min at 72°C.

### 2.4. Controlled Pollinations

In order to elucidate the expression profiles after pollination of the *PbS*_*26*_ *-RNase* gene, self-pollination (“Hongpisu” × “Hongpisu”) and cross-pollination (“Hongpisu” × “Jinhua”) tests were performed using 30 flowers with three replicates for each pollination combination. Anthers were collected from flowers at the BBS and incubated for 30 h to collect the pollens. For each pollination combination, flowers were emasculated, pollinated, and covered with paper bags to avoid contamination. In order to monitor pollen tubes growth in the style, samples were taken at 2, 8, 12, and 24 h after pollination and fixed in FAA solution. After fixation, the flowers were maintained in 70% ethanol and stored at 4°C until the time of pollen tubes growth was observed using a fluorescence microscope. According to the pollen tube growth, styles at 12 h and 24 h after pollination were collected for quantitative RT-PCR (qPCR) analysis. In addition, the percentages of fruit set and the number of full seeds were recorded four months after pollination. The fruit set percentage of below 30% was considered as incompatible [7].

### 2.5. Semiquantitative RT-PCR

The expression patterns of the *PbS*_*26*_ *-RNase *gene in different tissues/organs were studied by semiquantitative RT-PCR (semi-qPCR), in which pear *Actin *gene (GenBank accession number GU830958) was used as the reference gene. The *PbS*_*26*_ *-RNase* gene was amplified with primer pair S26PF1 (5′-GAATACAACCTTGAATTCTACTAA-3′) and S26PR1 (5′-CACAGCTGCCATGTTTGTT-3′), which could produce a fragment of 131 bp. The pear *Actin *gene was amplified with primer pair ActinF (5′-TGGTGTCATGGTTGGTATGG-3′) and ActinR (5′- CAGGAGCAACACGAAGTTCA-3′), which could produce a fragment of 173 bp. The reaction volumes were 20 *μ*L containing 1×PCR buffer, 0.5 mM dNTPs, 1.5 mM MgCl_2_, 0.5 mM of each primer, 1U Taq polymerase, and the 2.0 *μ*L synthesized cDNA first strand as template. The amplification program consisted of 35 cycles of 30 s at 94°C, 30 s at 58°C, 3 min at 72°C, and a final extension of 7 min at 72°C.

### 2.6. Quantitative RT-PCR

Quantitative RT-PCR (qPCR) was carried out with three replicates in each reaction using the BIO-RAD CFX system (Bio-Rad). *PbS*_*26*_ *-RNase* gene-specific primers, reference gene, and its corresponding primers were the same as described in semi-qPCR. PCR was performed in a 20 *μ*L volume containing 2 *μ*L diluted cDNA, 250 nM each primer, and 1 × SYBR Premix Ex Taq II (TaKaRa) using the same conditions with semiquantitative RT-PCR. The results from *PbS*_*26*_ *-RNase *gene-specific amplification were analyzed using the comparative Cq method which uses an arithmetic formula, 2^-ΔΔCq^, to obtain results for relative quantification [26].

### 2.7. Development of the Rapid Method for the *PbS* ****_***26***_ **** *-RNase* Gene Detection

To specifically detect the S_26_-allele, a primer pair S26PF2 (5′-GCACAGGAAATGACCCATC-3′) and S26PR2 (5′-GGTTCGATCGAGTACGTTG-3′) was designed via multiple genomic DNA sequence alignment that would produce a fragment of 254 bp by genomic PCR. The S26PF2 was located in intron region, and the S26PR2 was located between conserved 4 region (C4) and conserved 5 region (C5). The amplification program consisted of 35 cycles of 30 s at 94°C, 30 s at 53°C, 3 min at 72°C, and a final extension of 7 min at 72°C. The amplification products were detected by 1.5% agarose gel electrophoresis.

### 2.8. Cloning, Sequencing, and Sequence Analysis of PCR Products

The purified PCR products were TA-ligated into pMD18-T Vector (Takara, Japan) and transformed into *Escherichia coli* (*E. coli*) strain DH5*α*. The positive clones are screened by the white/blue colony and identified by plasmid PCR with BcaBESTTM sequencing primer M13-47 (5′-CGCCAGGGTTTTCCCAGTCACGAC-3′) and BcaBESTTM sequencing primer RV-M (5′-GAGCGGATAACAATTTCACACAGG-3′). PCR reactions were conducted with the following cycling conditions: one initial step of 4 min at 94°C, 35 cycles of 30 s at 94°C, 30 s at 62°C, 1.5 min at 72°C, and one final extension step of 10 min at 72°C. Target clones were sequenced in both directions by Shanghai Biosune Biotechnology Ltd.

Sequence data were analyzed with DNAMAN (version 6.0.40). Prediction of isoelectric point (pI), molecular weight (Mw), and Prosite was performed by using online ExPASy proteomics tools (http://expasy.org/tools/pi_tool.html). The BLAST algorithm was used to search the NCBI GenBank (http://www.ncbi.nlm.nih.gov/) databases for homologous sequences. Motif analysis of the target gene was conducted by Motif Scan (http://myhits.isb-sib.ch/cgi-bin/motif_scan) and sequence comparisons. Multiple amino acid sequences alignment was performed using the DNAMAN software. Based on this alignment, a phylogenetic tree was generated. The following S-RNase sequences, together with the *PbS*_*26*_ *-RNase* sequence, were used for the phylogenetic analysis: *P. bretschneideri*: PbS_13_-RNase (DQ414812), PbS_16_-RNase (DQ991388), and PbS_34_-RNase (DQ414813); *P. communis*: PcSa-RNase (AB236430), PcSb-RNase (AB236429), PcSd-RNase (AB236427), PcSh-RNase (AB236431), PcSk-RNase (AB236432), PcSq-RNase (AB236424), and PcSr-RNase (AB236426); *P. pyrifolia*: PpS_1_-RNase (AB002139), PpS_2_-RNase (D49527), PpS_3_-RNase (AB002140), PpS_4_-RNase (D49528), PpS_5_-RNase (D88282), PpS_6_-RNase (AB002142), PpS_7_-RNase (AB002143), PpS_8_-RNase (AB104908), and PpS_9_-RNase (AB104909); *M. domestica*: MdS_4_-RNase (AF327223), MdS_10_-RNase (AF327221), MdS_24_-RNase (AF016920), MdS_26_-RNase (AF016918), and MdS_31_-RNase (DQ135990); *P. armeniaca*: ParSc-RNase (DQ422947); *P. avium*: PavS_12_-RNase (AY259115), PavS_13_-RNase (DQ385842), PavS_23_-RNase (AY259114), PavS_24_-RNase (AY259112), and PavS_25_-RNase (AY259113); *P. dulcis*: PdSa-RNase (AB026836), PdSb-RNase (AB011469), PdSc-RNase (AB011470), and PdSd-RNase (AB011471); *P. salicina*: PsSa-RNase (AB026981), PsSb-RNase (AB026982), PsSc-RNase (AB084102), and PsSd-RNase (AB084103).

## 3. Results and Discussion

### 3.1. Sequence and Characterization of Full Length cDNA of the *PbS*_***26***_ *-RNase*

Previously, the *PbS*_*26*_ *-RNase* allele was identified in *P. bretschneideri* cultivar “Hongpisu” (S_12_S_26_) [13]. Its partial genomic sequence was isolated, but the functional completed cDNA has not been gained. The most common way to isolate full length cDNA includes the processes of 5′ RACE and 3′ RACE. The process of 5′ RACE is a more complicated and challenging procedure than 3′ RACE, with many more steps. The PF2 primer anneals to a conserved region in the 5′ untranslated region (UTR) of Japanese pear S-RNases [6]; accordingly the produced target gene contains initiation codon ATG in theory. Following RACE-PCR with primer PF2 and AUAP in this study, an approximately 900 bp product was yielded. Since the PF2 does not match the S_12_-RNase [27], the 900 bp product was considered to correspond to the *PbS*_*26*_ *-RNase *gene. The target clone was sequenced and confirmed as a full length cDNA encoding S_26_-RNase by detailed sequence analysis.

**Figure 1:**
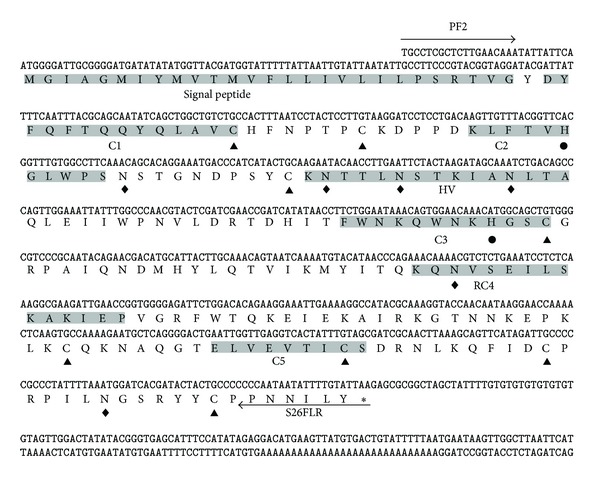
Nucleotide and deduced amino acid sequences of the full length *PbS*_*26*_ *-RNase* cDNA. The signal peptide, C1 to C5 domains, and HV region are shaded. Conserved cysteine residues, histidine residues essential for the RNase activity, and potential N-glycosylation sites are marked with symbols ▲, ●, and ◆, respectively, under the amino acid sequence. Asterisk indicates termination codon.

The cDNA sequence for *PbS*_*26*_ *-RNase* was 906 bp long with a complete open reading frame (ORF) of 684 bp encoding 228 amino acids (GenBank accession number EU081888). The putative initiation codon ATG was located at poisons 29–31. The termination codon TAA was present at positions 713–715, followed by 3′ UTR. The deduced amino acid sequences for the *PbS*_*26*_ *-RNase* had similar structural characteristics of pear S-RNases, for example, a signal peptide, five conserved regions, and a HV region. The molecular weight (Mw) and the isoelectric point (pI) of the *PbS*_*26*_ *-RNase* were predicted to be 26.2 kDa and 9.14, respectively, which agreed with the basic properties of pear S-RNases [7]. The *PbS*_*26*_ *-RNase* showed the conserved motifs with two histidine residues, His-60 and His-116, which are essential for T2/S type RNase activity [28]. It also harbored eight cysteine residues (Cys-42, Cys-49, Cys-75, Cys-119, Cys-183, Cys-198,Cys-209, and Cys-221) that were mostly conserved in S-RNases. These eight cysteine residues could form four disulfide bridges, which plays a significant role in the formation or stabilization of their tertiary structure [7]. The *PbS*_*26*_ *-RNase* also presented six potential N-glycosylation sites, of which only one, that is, Asn-, has glycans which were considered to be important for the folding and the stabilization of the core structure (Figure 1) [7].

### 3.2. Amino Acid Sequences Comparison and Phylogenetic Analysis

**Figure 2:**
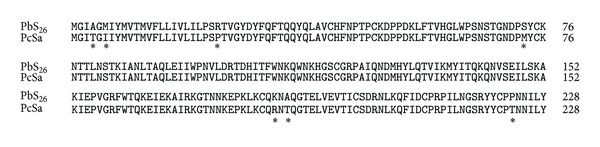
Alignment of amino acid sequences of the *PbS*_*26*_ *-RNase* and the PcSa-RNase. Amino acid differences are indicated by asterisk.

The newly cloned *PbS*_*26*_ *-RNase *gene allows us to better understand the evolutionary relationship of rosaceous S-RNases. The multiple amino acid sequences alignment was made using clustal X program with DNAMAN. A total of 39 complete amino acid sequences of S-RNases of eight species, including sweet cherry (*P. avium*), apricot (*P. armeniaca*), almond (*P. duslics*), plum (*P. salicina*), Japanese pear (*P. pyrifolia*), Chinese white pear (*P. bretschneideri*), European pear (*P. communis*), and apple *(M. domestica*), were used in the alignment. Among the *P. bretschneideri* species, the *PbS*_*26*_ *-RNase* showed the lowest similarity (64.0%) with the PbS_13_-RNase and the highest (90.4%) with the PbS_16_-RNase. However, the *PbS*_*26*_ *-RNase* showed extremely high similarities to *P. communis* PcSa-RNase (96.9%), which were remarkably higher than to *P. bretschneideri* S-RNases. Amino acid comparison between the *PbS*_*26*_ *-RNase* and the PcSa-RNase showed that only seven amino acids differences were presented in the two S-RNases (Figure 2). The existence of such two S-alleles, in one case, agreed with the hypothesis that new S-alleles are generated by an accumulation of point mutations of earlier-formed alleles, which may or may not lead to differences in amino acid sequence [29]. In another case, the two genes also may generate from the same ancestor after separate mutations.

**Figure 3:**
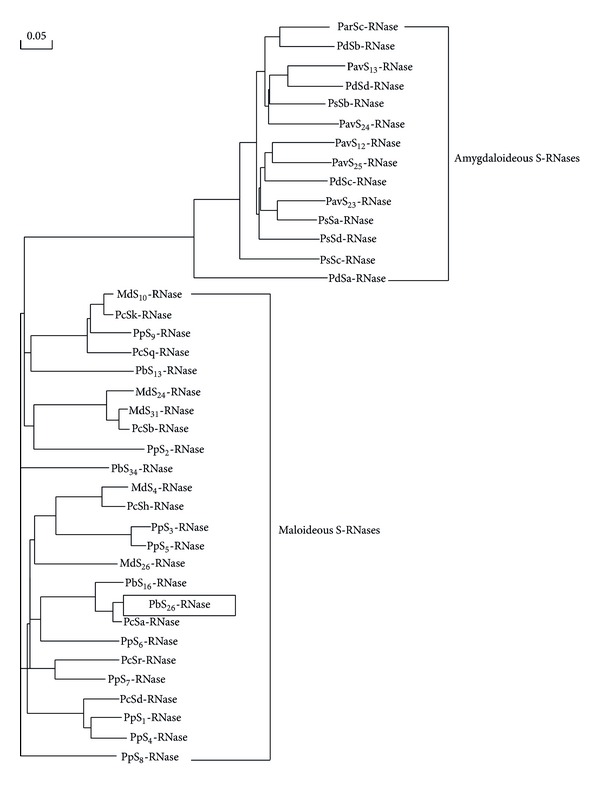
Phylogenetic analysis of the members of* S-RNase* genes in rosaceous plants. The *PbS*_*26*_ *-RNase* is boxed.

Based on the multiple sequence alignment, a phylogenetic tree was generated (Figure 3). As indicated by the tree, the 39 S-RNases were divided into two subfamily-specific groups, that is, amygdaloideous S-RNases and maloideousS-RNases. The amygdaloideous group includes S-RNases of pears (*P. pyrifolia*, *P. bretschneideri*, and *P. communis*) and apple (*M. domestica*). The maloideous group includes those of almond (*P. duslics*), apricot (*P. armeniaca*), sweet cherry (*P. avium*), and plum (*P. salicina*). In the maloideous group, pear and apple S-RNases were closely related that they did not form species-specific subgroup. In the case of amygdaloideous group, the almond, apricot, plum, and sweet cherry S-RNases were grouped with one another and did not form species-specific subgroup either. The phylogenetic analysis of the study agreed with the proposal of Ushijima et al. [30]; namely, the divergence of S-RNase alleles predated speciation of the Rosaceae family, but it occurred shortly after the divergence into subfamilies.

### 3.3. The *PbS*_***26***_ *-RNase* Gene Was Specifically Expressed in the Style

**Figure 4:**
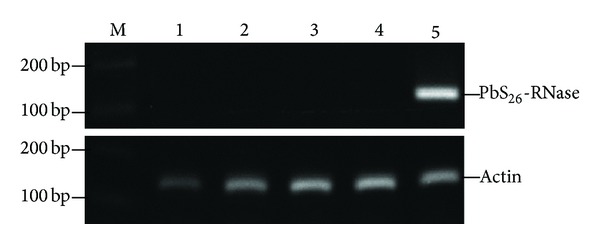
Semi-qPCR for the *PbS*_*26*_ *-RNase* gene expression detection in different tissues/organs. 1–5: roots, stems, leaves, stamens, and styles.

To investigate tissues/organs expression of *PbS*_*26*_ *-RNase* gene, semi-qPCR was performed on total RNA of styles prior to pollination, stamens, roots, stems, and leaves of “Hongpisu” pear. Following RT-PCR with *PbS*_*26*_ *-RNase* gene-specific primers, one fragment of expected sized (130 bp) was produced from styles, but not from the other tissues/organs (Figure 4). The results suggested that the *PbS*_*26*_ *-RNase* gene was specific in pears. The expression patterns coincided with those of other *S-RNase* genes in Rosaceae plants such as apple [31], Japanese pear [7], sweet cherry [32], and Japanese apricot [33].

### 3.4. Comparison of Expression Patterns of the *PbS*_***26***_ *-RNase* Gene between Self-Pollination and Cross-Pollination

Although many data show that the *S-RNase *genes are specific in the style, little is known about the expression profiles of the pear S-alleles during the style development, including before and after pollination. In this study, qPCR indicated that the *PbS*_*26*_ *-RNase* gene showed an increased expression pattern in the developing style before pollination in “Hongpisu.” A slight transcript level of the Pb_26_-RNase allele was detected at 96 h BA, and then it rapidly increased at 48 h BA and at BBS (Figure 7).

**Figure 5:**
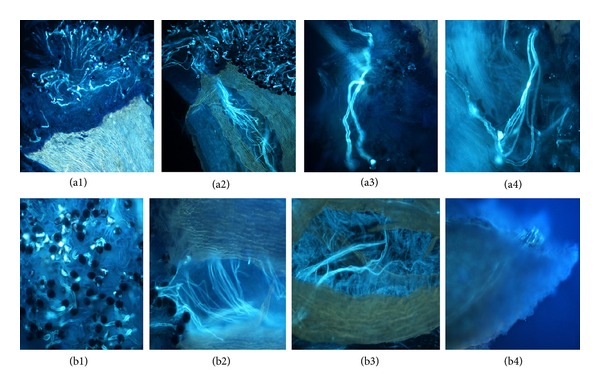
Fluorescence micrographs of squashed styles of “Hongpisu” harvested after different pollination treatments. (a1) Germination of pollen grains on stigma 2 h after self-pollination. (a2) Growth of pollen tubes in style transmitting tissue 6 h after self-pollination. (a3) Growth inhibition of pollen tubes in style transmitting tissue 8 h after self-pollination. (a4) Twisted pollen tubes 12 h after self-pollination. (b1) Germination of pollen grains on stigma 1 h after cross-pollination. (b2) Growth of pollen tubes in style transmitting tissue 8 h after cross-pollination. (b3) Growth of pollen tubes in style transmitting tissue 12 h after cross-pollination. (b4) Pollen tubes arrive at the base of the style 24 h after cross-pollination.

**Figure 6:**
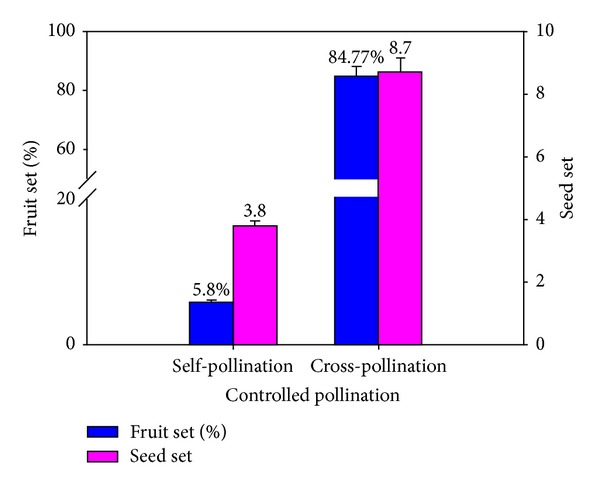
Percentages of fruit set and seed set of “Hongpisu” after controlled pollination.

To further investigate the expression patterns after compatible pollination and incompatible pollination and compare their difference, controlled pollinations, that is, “Hongpisu” × “Hongpisu” and “Hongpisu” × “Jinhua,” were conducted. In self-pollination, pollen tubes were inhibited and twisted 12 h after pollination (Figure 5, a4). The self-pollination led to the low percentages of fruit set (5.8%) and seed set (on the average 3.7 seeds per fruit) (Figure 6), showing incompatible behaviors. In cross-pollination, in contrast, pollen tubes could grow to the cut end of the style, that is, the stylar base 24 h after pollination (Figure 5, b4). The cross-pollination resulted in remarkably higher percentages of fruit set (84.6%) and seed set (on the average 8.7 seeds per fruit) (Figure 6), showing compatible behaviors.

**Figure 7:**
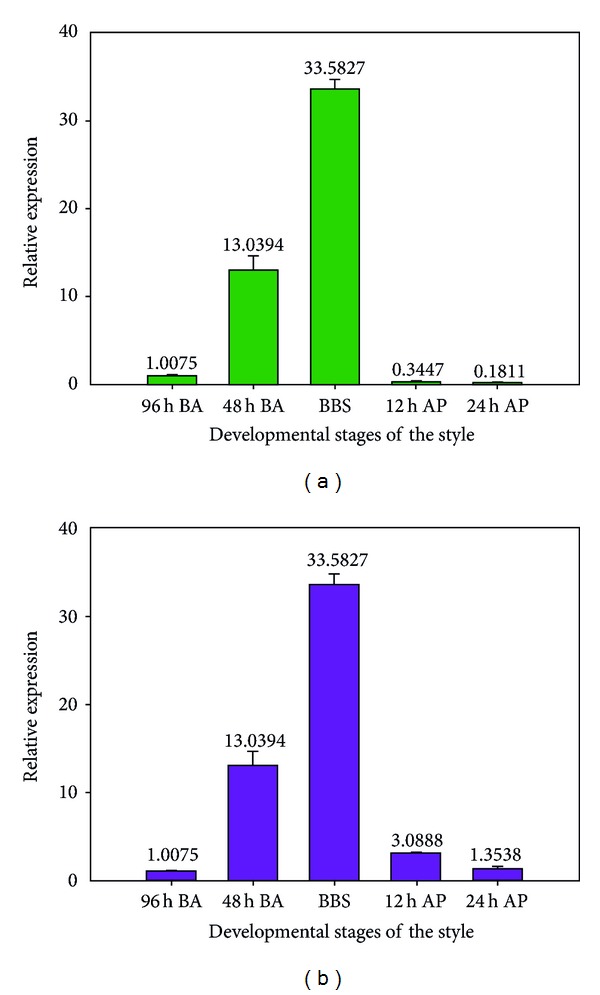
QPCR analysis for the *PbS*_*26*_ *-RNase *gene expression detection during styles development in “Hongpisu.”  (a) Relative expression of the *PbS*_*26*_ *-RNase *gene in the style before and after self-pollination; (b) relative expression of the *PbS*_*26*_ *-RNase *gene in the style before and after cross-pollination. BA: before anthesis; BBS: bell balloon stage; AP: after pollination.

After pollination, the expression level of *PbS*_*26*_ *-RNase* allele gradually decreased in the style of both different pollinations (Figure 7). However, the expression level of the *PbS*_*26*_ *-RNase *gene was higher after compatible pollination than after incompatible pollination, with the abundance of transcript being around 9-fold and 7-fold higher at 12 h AP and 24 AP, respectively, in cross-pollination than in self-pollination. This is the first report of the expression patterns of pear *S-RNase *genes in the developing style after pollination. The difference of *S-RNase *gene expression level between self-pollination and cross-pollination may be associated with the behaviors of different pollen S-genes. The pollen S-gene is identified as an F-box protein (SFB/SLF) which appears to act as a component of SCF complex. The SCF complex is associated with 26S proteasome degradation pathway in which non-self-S-RNases are targeted for degradation, and then the self-S-RNases are retained to specifically inhibit the growth of self-pollen tubes [34]. More recently, Xu et al. reported that two pollen specific SLF-interacting Skp1-like (SSK) proteins, PbSSK1 and PbSSK2, in* P. bretschneideri* could connect PbSLFs to PbCUL1 to form a putative canonical SCF^SLF^ (SSK/CUL1/SLF) complex [24]. Based on the studies of Qiao et al. [34] and Xu et al. [24], we presume that pollen specific SFB_26_/SLF_26_ may be involved in the formation of 26S proteasome pathway and specifically degrade the non-self-S-RNases after pollination, resulting in the fact that the *PbS*_*26*_ *-RNase* is reserved. Therefore, the abundance of the *PbS*_*26*_ *-RNase *gene transcript in the style could be greater after cross-pollination than after self-pollination.

### 3.5. Development of the Rapid Method for Identifying the *PbS*_***26***_ *-RNase* Gene

**Figure 8:**
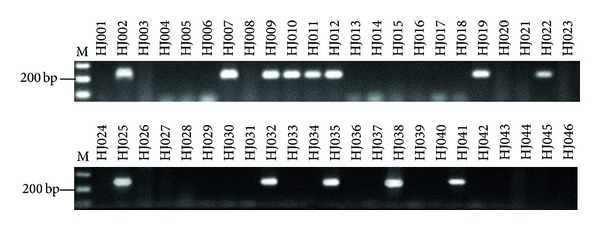
Amplification of S-RNase alleles in 46 progenies of “Hongpisu” × “Jinhua” with S26PF2 and S26PR2 primers. M: 100 bp DNA ladder; lanes 1 to 46 correspond to the progeny numbers.

In Japanese pear, a PCR-RFLP system was established for S-genotype assignments based on cloning of *S*_*1*_ *- to S*_*9*_ *-RNase *gene [5, 7]. Herein, we reported a different method for rapidly identifying *S-RNase *genes in Chinese pears. For the *PbS*_*26*_ *-RNase *gene, a novel primer pair S26PF2 and S26PR2 was designed based on DNA sequence comparisons. Genomic DNA of “Hongxiangsu” was amplified with S26PF2 and S26PR2, and one specific fragment with expected size (254 bp) was generated. Sequencing of this fragment revealed that it corresponded to the *PbS*_*26*_ *-RNase* allele. Based on the *PbS*_*26*_ *-RNase* allele, the other one allele was easily identified as S_21_ by further PCR amplification and DNA sequencing. The S26PF2 and S26PR2 primer combination was also applied in 46 progenies of “Hongpisu” (S_12_S_26_) × “Jinhua” (S_13_S_18_), and the 254 bp sized fragment was produced in 13 progenies (Figure 8), suggesting that the 13 individuals harbor the *PbS*_*26*_ *-RNase* allele. These results showed that the newly designed PbS_26_-specific primers could be used for rapid identification of the *PbS*_*26*_ *-RNase* allele and will be useful in pear breeding programs.
